# Stimulation of Heavy Metal Adsorption Process by Using a Strong Magnetic Field

**DOI:** 10.1007/s11270-017-3672-2

**Published:** 2018-01-05

**Authors:** Krzysztof Rajczykowski, Krzysztof Loska

**Affiliations:** Institute of Water and Wastewater Engineering, Faculty of Energy and Environmental Engineering, Silesian University of Technology, Konarskiego 18, 44-100 Gliwice, Poland

**Keywords:** Heavy metal removal, Heavy metal adsorption, Magnetic field modification, Adsorption modification

## Abstract

The adsorption process is one of the most important techniques of water and wastewater treatment technology. Therefore, there are many methods allowing to improve the effectiveness of these processes based mainly on the chemical modification of adsorbents. However, they are always associated with the necessity of introducing an additional wastes or sewage to the environment. That is why a purpose of the presented was to investigate an innovative and noninvasive adsorption supporting method based on the using of a static magnetic field. The results showed that in the adsorption process of equimolar copper, nickel, and cadmium mixture, a presence of the magnetic field may increase the effectiveness of the process, with respect to copper by more than 40% and a summary molar removal was increased about 11%. However, the effectiveness of the analyzed modification depends largely on the heavy metal equilibrium concentration, and when it increases, a beneficial effect of magnetic field significantly decreases. Nevertheless, due to the fact that heavy metal adsorption processes are very important part of environmental engineering technologies, it can be assumed that further work on magnetic modification of these processes can allow for a significant improvement of many water and wastewater purification plants.

**Figure:**
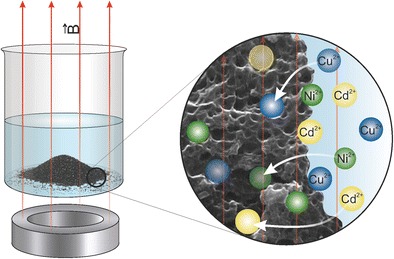
Graphical Abstract

Graphical Abstract

## Introduction

Heavy metal pollution of the environment is one of the most important problems of the modern environmental engineering, and the main sources of those pollutions are different types of industrial wastewaters (Nagajyoti et al. 2010; Fu and Wang 2011). Therefore, there are many different methods of removing this type of contaminants from water and wastewater. One of these methods is the adsorption process in which heavy metal ions are removed from the solution by depositing them directly on the surface of the specially chosen adsorbent. A great advantage of adsorption methods is that various types of wastes can be used as an adsorbent, such as wastes from the agriculture or food industry like e.g., straw and coconut shells. This, in turn, allows for a significant reduction of the cost of the process; however still, the most effective group of the adsorbents is activated carbons, generated by the thermal treatment of different types of charcoal (Bansal and Goyal 2005). Nowadays, there are many different methods of increasing the efficiency of adsorbents, which are based mainly on the specially selected chemical modifications (Singha and Guleria 2014). However, a significant drawback of these methods is an often need of using aggressive chemicals, which in turn leads to production of the additional quantity of dangerous wastes and wastewaters. Therefore, an aim of the presented study was to investigate a novel, noninvasive, and environmental friendly modification of the adsorption process by treating the reactor wherein a process takes place by a strong external magnetic field. In the scientific literature, there are many reports concerning the changes of physicochemical properties of water and aqueous solutions caused by the magnetic field (Pang and Deng 2008; Cai et al. 2009; Ambashta and Sillanpää 2010). In their work, Cai et al. demonstrated that in the samples exposed to a strong magnetic field, with magnetic induction value 0.5 T, a surface tension of the samples was significantly reduced, while the viscosity increased (Cai et al. 2009). In addition, it is was also noted that using a strong magnetic fields may reduce the strength of hydrogen bonds present in the magnetized water samples (Wang et al. 2013). It is worth to notice that there was also a several studies related to the influence of magnetic field on the adsorption processes, such as methyl blue (Xiaolong et al. 2012) or methylene blue (Guoting et al. 2016) adsorption processes. In both cases, it has been shown that exposition of the processes to the external magnetic field leads to the significantly increase of the adsorption process efficiency. Beneficial effects of the external magnetic field was also found for some other processes of chemical engineering, such as ammonia absorption (Xiaofeng et al. 2007), crystallization processes (Tai et al. 2008), and biological sewage treatment process (Liu et al. 2008; Tomska and Wolny 2008). Nevertheless, in the case of heavy metal adsorption processes, the potential influence of the magnetic field is still unknown, and taking into account that they are one of the main groups of pollutants removed by the adsorption methods, it seems that this gap in the current state of knowledge needs to be completed.

An important problem related to these phenomena is the fact that at the present day, there is no single and coherent theory which would satisfactorily explain the mechanism of magnetic field influence on the mentioned chemical and environmental engineering processes. Of course, it does not mean that over the years, such attempts were not made at all, but only that existing explanations have rather phenomenological and particular character, which does not allow to use them to predict a mentioned influence on the other processes. So, depending on the author and type of the analyzed process, there are theories trying to explain observed intensification effects of some chemical engineering processes, basing on the impact of classical Lorentz force on the charged particles. However in a case of that explanation, it is good to remember that Lorentz force is a typical conservative force, so it cannot change the internal or free energy values of the particles, so thus any changes in the energy balance of the process also seem to be quite unlikely. On the other hand, *Farmanzadeh and Tabari*, in their extensive work dedicated to the theoretical analysis of the electric field effect on adsorption processes at the surface of carbon nanotubes, postulate that the ability to effectively modify of process is a result of changes in the structure of HOMO and LUMO orbitals of the adsorbent particles (Farmanzadeh and Tabari 2013). Although their analysis was related to changes caused not by the magnetic but an electric field, a numerous of similarities between these two components of the electromagnetic field makes that it seems possible to apply a similar approach to the problems of magnetic field influence.

## Materials and Methods

Heavy metal solutions used during the study were prepared by dissolving an appropriate amount of nickel, cadmium, and copper nitrates in the demineralized water. All of the chemical reagents were analytical pure in order to avoid possible errors due to sample impurities. The adsorption process was conducted in a 400-cm^3^ volume glass reactor by the addition of 0.5 g of activated carbon in the form of fine granule size of 1–4 mm (*extra-ChemWD/w by Chempur*) to a 200 cm^3^ of the initial heavy metal solution. The magnetic field analyzed during the experiment was generated by a permanent neodymium ring magnets type N38 with dimensions of 64/32/25 mm (od/id/thick). The coercivity value of the magnets was equal to 955 kA/m and their energy density was about 295 kJ/m^3^. Magnetic induction of the magnets was 0.517 T (measured at a distance of 0.7 mm from the surface of the magnet along to the magnetization axis). Next, the reactors were placed in a special rack, allowing for the simultaneous shaking of the six reactors in the same time, along with the possibility of mounting of mentioned neodymium magnets, directly under the bottom of the reactors. The adsorption processes both modified and unmodified were carried out with six repetitions and lasted 60 min, because it was considered as an optimal reaction time, due to the results obtained in preliminary studies and during the adsorption kinetics analysis. In addition, an effectiveness of the adsorption process is strongly dependent on the pH value of the reaction mixture; therefore, during the preliminary studies, it was investigated that within pH value, efficiency of the process would be the best. Obtained results indicate to a comparable efficiency of the process, observed at the pH at 5 and 7; however, in pH 7, a solution became turbid, which may suggest that the heavy metals began to precipitate from the solution due to a reduction of their solubility in higher pH. That is why all of the further analysis during the study was conducted in pH value equals to 5. The concentration of heavy metals in the initial and final solutions was analyzed by using atomic absorption spectroscopy on a flame spectrophotometer SpectrAA 880 by Varian.

Results obtained during the tests were also used to find a proper adsorption model, according to which the process took place. For this purpose, common adsorption isotherm models were analyzed, and their parameters and R^2^ coefficient was determined on the basis of nonlinear regression methods using the *SciDAVis* software. The table below shows all adsorption models that were analyzed during the research and their equations of which were used for the fitting (Table 1).

**Table 1:** Isotherm adsorption models, and their equation, used during the study to fit the model to obtained experimental results

Type of isother	Isotherm equation	Reference
Langmuir	${q}_e=\frac{q_{max}\ast b\ast {C}_e}{1+b\ast {C}_e}$	(Syers et al. 1973)
Freundlich	${q}_e={K}_F\ast {C}_e^{\left(\frac{1}{n}\right)}$	(Sposito 1980)
Redlich-Peterson	${q}_e=\frac{K_{RP}\ast {C}_e}{1+{a}_R\ast {C}_e^{\beta }}$	(Wu et al. 2010)
Langmuir-Freundlich	${q}_e=\frac{q_{max}{\left({K}_a\ast {C}_e\right)}^n}{{\left({K}_a\ast {C}_e\right)}^n+1}$	(Jeppu and Clement 2012)
Temikn	${q}_e=\frac{R\ast T}{b}\ln \left({A}_T\ast {C}_e\right)$	(Dada et al. 2012)

Moreover, in order to confirm the statistical significance of the magnetic field influence on heavy metal adsorption process, an ANOVA analysis was performed.

## Results and Discussion

### Adsorption Kinetic Analysis

In order to determine the optimum reaction time and type of kinetics according to which the adsorption of selected heavy metals on activated carbon takes place, the appropriate kinetic model’s parameters were calculated (Table 2).

**Table 2:** Basic parameters of pseudo-first- and second-order kinetic models for each kind of adsorbed heavy metals and their mixture

Type of metal	Modification	q_e_ [mmol/g]	Pseudo-first order		Pseudo-second order	
			k_1_ [1/min]	*R* ^2^	k_2_ [mmol/g*min]	*R* ^2^
Copper	Unmodified	0.0291	0.0325	0.992	0.7080	0.900
	Magnetic modification	0.0340	0.0362	0.988	0.5261	0.300
Nickel	Unmodified	0.0134	0.0357	0.972	3.0241	0.985
	Magnetic modification	0.0111	0.0366	0.929	6.9596	0.983
Cadmium	Unmodified	0.0277	0.0357	0.902	3.3433	0.984
	Magnetic modification	0.0290	0.0336	0.991	2.5426	0.980
Sum	Unmodified	0.0593	0.0341	0.996	0.5732	0.990
	Magnetic modification	0.0623	0.0394	0.953	0.5710	0.996

On the basis of data, presented in the table above, an important observation related to reaction kinetics can be made. First of all, *R*^2^ correlation coefficients show that the studied processes of heavy metals adsorption, both in the presence and absence of a magnetic field, were suitable rather to the pseudo-first-order kinetics model than to the second-order. In addition, the k_1_ kinetics constant value was higher for processes modified by using the external magnetic field for all of the analyzed metals except cadmium, which in turn means that proposed modification method can be a very effective way to increase a rate of the heavy metal adsorption process. Moreover, a q_e_ value that specifies the maximum removal of heavy metals was also higher in the case of the reaction systems treated by a magnetic field.

### Magnetic Field Influence on Adsorption Efficiency

**Fig. 1:**
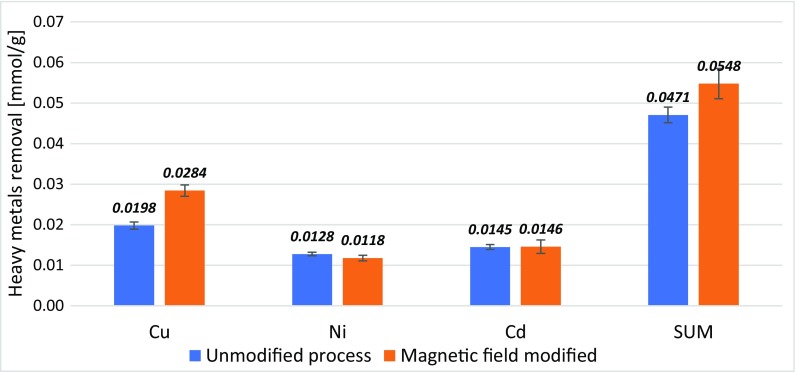
Molar removal of selected heavy metals forms the solution with initial concentration 0.05 mmol/dm^3^ for each of the metal

As a result of the conducted study, it was showed that the external magnetic field can significantly contribute to increasing the efficiency of selected heavy metal adsorption process from aqueous solutions (Fig. 1).

Data presented in the figure refer to the adsorption process from solution with an initial concentration of all heavy metals equal to 0.05 mmol/dm^3^. It is worth to notice that the difference in copper removal efficiency was high enough, to ensure a higher general molar removal of heavy metals from the solution for the magnetic modified reaction systems. However, an observed impact is largely dependent on both the type of removed metals and their equilibrium concentration. It was noted that in the case of copper, nickel, and cadmium mixture, a beneficial effect of magnetic field was observed primarily for copper adsorption, and in turn, the differences in cadmium and nickel removal were relatively low. When the heavy metal concentrations in the solution were higher, the differences between modified and unmodified adsorption decreased to finally can be regarded as negligible for processes conducted for solutions with initial concentrations 1 mmol/dm^3^. Results for all of the analyzed initial concentration are presented in Table 3.

**Table 3:** Comparison of heavy metals removal in systems unmodified and modified with magnetic field in different initial concentration of heavy metals

Metal	Modification type	Heavy metal removal in mmol/g at the different initial concentrations [mmol/dm^3^]				
		0.075	0.15	0.3	0.6	1
Copper	Unmodified	0.0076 (± 0.0006)	0.0198 (± 0.0008)	0.0418 (± 0.0012)	0.0518 (± 0.0014)	0.0521 (± 0.0022)
	Magnetic field	0.0116 (± 0.0005)	0.0271 (± 0.0031)	0.0435 (± 0.0008)	0.0526 (± 0.0031)	0.0507 (± 0.0027)
Nickel	Unmodified	0.0661 (± 0.0016)	0.0639 (± 0.0021)	0.0548 (± 0.0035)	0.0537 (± 0.0043)	0.0244 (± 0.0136)
	Magnetic field	0.0595 (± 0.0011)	0.0599 (± 0.0037)	0.0485 (± 0.0023)	0.0514 (± 0.0089)	0.0196 (± 0.0103)
Cadmium	Unmodified	0.0555 (± 0.0008)	0.0725 (± 0.0015)	0.0698 (± 0.0059)	0.0731 (± 0.0051)	0.0725 (± 0.0227)
	Magnetic field	0.0601 (± 0.0015)	0.0727 (± 0.0039)	0.0675 (± 0.0125)	0.0751 (± 0.0048)	0.0615 (± 0.0146)
SUM	Unmodified	0.1598 (± 0.0018)	0.2353 (± 0.0025)	0.3337 (± 0.0076)	0.3859 (± 0.0043)	0.3574 (± 0.0327)
	Magnetic field	0.1778 (± 0.0017)	0.2683 (± 0.0064)	0.3333 (± 0.0127)	0.3895 (± 0.0084)	0.3344 (± 0.0166)

Analysis of the data presented in the table proves a high effectiveness of the proposed modification methods, for systems with the initial concentration of the heavy metal less than 0.3 mmol/dm^3^. In the case of higher heavy metal initial concentrations, the influence of magnetic field on the adsorption processes became to be negligibly small. Additionally, in order to better characterize the adsorption processes, the attempts were made to match an obtained experimental results to one of the commonly used adsorption models such as Langmuir, Freundlisch, Langmuir-Freundlisch, Redlich-Peterson, and Temkin isotherm adsorption model. However, it was found that the best fit was achieved in the case of Langmuir and Langmuir-Freundlisch adsorption model. In addition, in order to increase the reliability of the model, for cases of summary removal of all three metals from the mixture, a multi-component Langmuir isotherm was used instead of the classical simple Langmuir isotherm. All of the basic parameters of these two mentioned models were presented in the table below (Table 4).

**Table 4:** Parameters of basic isotherm adsorption models for each of the metals

Type of metal	Modification	Langmuir			Langmuir-Freundlich			
		q_max_	b	*R* ^2^	q_max_	K_a_	n	*R* ^2^
Copper	Unmodified	0.0697	4.65	0.876	0.0529	8.092	2.525	0.999
	Magnetic modification	0.0619	8.37	0.897	0.0517	11.945	2.188	0.978
Nickel	Unmodified	0.0526	− 9.59	0.333	0.0623	1.091	− 3.598	0.886
	Magnetic modification	0.0478	− 1.44	0.333	0.0562	1.097	− 5.026	0.851
Cadmium	Unmodified	0.0752	62.83	0.631	0.0723	24.21	3.562	0.928
	Magnetic modification	0.0698	178.81	0.138	0.0712	1.923	−0.039	0.343
Sum	Unmodified	0.420	3.88	0.925	0.379	4.296	1.659	0.957
	Magnetic modification	0.394	5.84	0.829	0.361	5.534	1.974	0.863

Analysis of the data presented in the table leads to the conclusion that the application of an external magnetic field may slightly reduce the theoretical maximum adsorption capacity of the adsorbent. However, it should be also noted that for the modified system, *R*^2^ determination coefficient was significantly lower. It is probably caused by a presence of the additional factors related with magnetic field that are not included in the equations but can have an influence on the shape and course of adsorption isotherms. Therefore, it seems necessary to develop a new, appropriate model of adsorption isotherms that would include this type of interaction and thus allow to predict the theoretical maximum adsorption capacity of the adsorption processes, modified with a strong external magnetic field. Finally, in order to verify the statistical significance of the obtained results, an ANOVA test was conducted for each of the metal and a summary removal results. Table below provides a detailed summary of the parameters of ANOVA analysis (Table 5).

**Table 5:** Results of ANOVA analysis for adsorption isotherm studies including *F*-statistic, *p* value, and sum of squares (SS) for each type of metals and their mixture

Parameter	Copper	Nickel	Cadmium	Sum
*F* value	13.84	4.953	0.206	0.951
*p* value	0.0005	0.031	0.652	0.334
SS	0.369	1.194	0.307	0.0002

To determine the statistical significance of the applied magnetic modification of the adsorption process, for each kind of metals and their mixtures, *F* values presented in the table above should be compared with a tabular critical *F* value. For the analyzed model, with two different groups (modified and unmodified), six repetition, and five different initial concentrations, at the significant level *α* = 0.05, a critical value of F-parameter is equal to *F*_(1,54)_ = 4.0195. Therefore, on the basis of presented results, it can be concluded that magnetic modification used in the study had a statistically significant effect on the copper and nickel adsorption processes. In turn, in case of cadmium and a mixture of all three metals, the hypothesis about the lack of differences between modified and unmodified systems cannot be rejected. For metal mixture that conclusion is quite unexpected, especially, due to the fact that the visual analysis of the data presented in Fig. 1 (even taking into account the standard deviation) seemed to confirm the existence of a mentioned influence of magnetic field. However, it is worth to notice that Fig. 1 shows only the results for the lowest of analyzed initial concentration values, and the ANOVA statistical analysis was carried out in regard to the whole range of initial concentrations. That is why, in the case of heavy metal mixture, an additional analysis was conducted, where the value of the *F*-statistic was calculated only for the lowest initial concentration (0.075 mmol/dm^3^) and it was equal to *F* = 3.338. Comparison of that value with tabular values of F-parameters shows that only after the increasing of a significant level to *α* = 0.1, for which the critical *F* value is equal to *F*_(1.10)_ = 3.285, the impact of the modification can be considered as statistically significant.

## Conclusions

A fundamental conclusion from the conducted research is that it is possible to speed up and increase the efficiency of the heavy metal adsorption process from the aquatic solutions if a proper term and conditions will be maintained, especially an initial concentration of metals in the solution. This is a very important discovery because an implementation of proposed magnetic modification does not require any additional chemicals that may adversely affect the environment or simply increase the amount of wastes and wastewaters generated during the process. This situation usually occurs with a chemical modification processes of the adsorbents; therefore, it should be considered a possibility of replacement those methods by magnetic modifications. An important issue in the further development of magnetic modification methods is the fact that despite numerous theories about the mechanism of the observed influence, there is still no unequivocal and universal answer to the question of how exactly magnetic fields can affect to these processes. The existence of that influence seems to be possible, according to the basic thermodynamic functions of the state like for example well-known formula for the free energy of the particle placed in the external magnetic field, which is given by (Couture and Zitoun 2000):

1 $$ dF=- SdT- MdB $$

where:

F: free energy of the particles, placed in the magnetic field
S: entropy of the system
B: induction of the external magnetic field
M: total magnetic moments of the system

On the basis of Eq. 1, it is clear that the presence of the magnetic field should provide a contribution to Helmholtz free energy of the particles placed in that field if the value of magnetic induction will be sufficiently high. However in the case of the adsorbent, it seems to be obvious that changes and rearrangements of the magnetic moments can significantly change the energetical structure and properties on the macroscopic scale, but in the case of free metal ions dissolved in the solution, this effect is not so clear. So thus it could be assumed that magnetic field can influence only to the adsorbents properties, but analysis of the obtained results shows clearly that different effects were obtained for the different kinds of metals. Moreover, copper was the only diamagnetic metals from the entire three heavy metals used during the study, and therefore, it seems possible that magnetic properties of the materials can somehow determine the susceptibility of the adsorbed metals in this kind of modifications. Another explanation can be a mentioned earlier theory, proposed by *Farmanzadeh and Tabari*, basing on the changes in the energetical structures of HOMO and LUMO orbitals in the adsorbent particles (Farmanzadeh and Tabari 2013). However, if the stimulating effect would refer only to the adsorbent particles, once again it would be difficult to explain why this effect is associated mostly with the copper, not the other metals adsorption. Therefore, it seems that somehow, the energy stored in the electric field has to affect not only to the adsorbent particles but also to heavy metal particles that are present in the solution.

In summary, although it is not entirely clear how exactly the magnetic field can influence the heavy metal adsorption processes, this method cannot be underestimated as a potential solution for increasing the effectiveness of those processes with respect to the selected compounds, especially due to the fact that it does not involve any further operating costs or generating additional contaminants to the environment.
